# Post-contrast acute kidney injury in a hospitalized population: short-, mid-, and long-term outcome and risk factors for adverse events

**DOI:** 10.1007/s00330-020-06690-3

**Published:** 2020-02-21

**Authors:** Wei Cheng, Xi Wu, Qian Liu, Hong-Shen Wang, Ning-Ya Zhang, Ye-Qing Xiao, Ping Yan, Xu-Wei Li, Xiang-Jie Duan, Jing-Cheng Peng, Song Feng, Shao-Bin Duan

**Affiliations:** 1 Department of Nephrology, The Second Xiangya Hospital, Hunan Key Laboratory of Kidney Disease and Blood Purification, Central South University, 139 Renmin Road, Changsha, 410011 Hunan China; 2 Information Center, The Second Xiangya Hospital, Central South University, Changsha, 410011 Hunan China; 3 Information Center, The Xiangya Hospital, Central South University, Changsha, 410008 Hunan China

**Keywords:** Contrast media, Acute kidney injury, Prognosis, Risk factors

## Abstract

**Objectives:**

To investigate the prognosis including major adverse kidney events within 30 days (MAKE30) and 90-day and 1-year adverse outcome in hospitalized patients with post-contrast acute kidney injury (PC-AKI) to identify high-risk factors.

**Methods:**

This retrospective observational study included 288 PC-AKI patients selected from 277,898 patients admitted to hospitals from January 2015 to December 2015. PC-AKI was defined according to the 2018 guideline of European Society of Urogenital Radiology. Multivariable Cox regression and logistic regression analyses were used to analyze main outcome and risk factors.

**Results:**

PC-AKI patients with AKI stage ≥ 2 had much higher incidence of MAKE30 than those with AKI stage 1 (RR = 7.027, 95% CI 4.918–10.039). Persistent renal dysfunction, heart failure, central nervous system failure, baseline eGFR < 60 mL/min/1.73 m^2^, oliguria or anuria, blood urea nitrogen ≥ 7.14 mmol/L, respiratory failure, and shock were independent risk factors of 90-day or 1-year adverse prognosis (*p* < 0.05). Compared with transient renal dysfunction, PC-AKI patients with persistent renal dysfunction had a higher all-cause mortality rate (RR = 3.768, 95% CI 1.612–8.810; RR = 4.106, 95% CI 1.765–9.551) as well as combined endpoints of death, chronic kidney disease, or end-stage renal disease (OR = 3.685, 95% CI 1.628–8.340; OR = 5.209, 95% CI 1.730–15.681) within 90 days or 1 year.

**Conclusions:**

PC-AKI is not always a transient, benign creatininopathy, but can result in adverse outcome. AKI stage is independently correlated to MAKE30 and persistent renal dysfunction may exaggerate the risk of long-term adverse events.

**Key Points:**

*• PC-AKI can result in adverse outcome such as persistent renal dysfunction, dialysis, chronic kidney disease (CKD), end-stage renal disease (ESRD), or death.*

*• AKI stage is independently correlated to MAKE30.*

*• Persistent renal dysfunction may exaggerate the risk of long-term adverse events.*

**Electronic supplementary material:**

The online version of this article (10.1007/s00330-020-06690-3) contains supplementary material, which is available to authorized users.

## Introduction

Iodine-based contrast media (ICM) are nephrotoxic and may account for a significant number of cases of hospital-acquired acute kidney injury (AKI) [1] or be responsible for the worsening of chronic kidney disease (CKD) [2]. The Contrast Media Safety Committee (CMSC) of the European Society of Urogenital Radiology (ESUR) [3] recommends that the term post-contrast acute kidney injury (PC-AKI) proposed by the American College of Radiology (ACR) [4] should replace the former term of contrast-induced acute kidney injury (CI-AKI), which is a correlative diagnosis instead of a causative diagnosis.

The CMSC, like the European Renal Best Practice (ERBP) working group, recommends that the definition of PC-AKI or CI-AKI should use the Kidney Disease: Improving Global Outcomes (KDIGO) definition of AKI [3, 5]. Recent progress in definitions has revived interest in the incidence and prognostic implications of PC-AKI. Its potential adverse effect on prognosis and addition to health care costs offer challenges in decision-making [6, 7]. We used to believe that most of the patients actually diagnosed as PC-AKI only presented with transient elevations in serum creatinine (SCr) level, which resolved spontaneously several days later. In fact, a significant part of the hospitalized population has developed into persistent renal dysfunction, resulting in adverse outcome, even end-stage renal disease (ESRD), or mortality [8–14]. The temporal evolution of renal function in patients with PC-AKI and the difference of adverse outcome between persistent renal dysfunction (RD) and transient renal injury need to be further studied.

Attention to the incidence and risk factors of major adverse kidney events (MAKE) recently increased [15, 16] in AKI patients while shifting the focus from short-term, surrogate measures [17] to long-term, more patient-centered endpoints [18, 19].

This retrospective study investigated the outcome of hospitalized patients with PC-AKI at 30 days (MAKE30, 30 days mortality, receipt of new renal replacement therapy (RRT) or persistent renal dysfunction), 90 days (CKD, mortality), and 1 year (ESRD, mortality). The aim was to identify high-risk factors.

## Method

### Study design and patient population

**Fig. 1:**
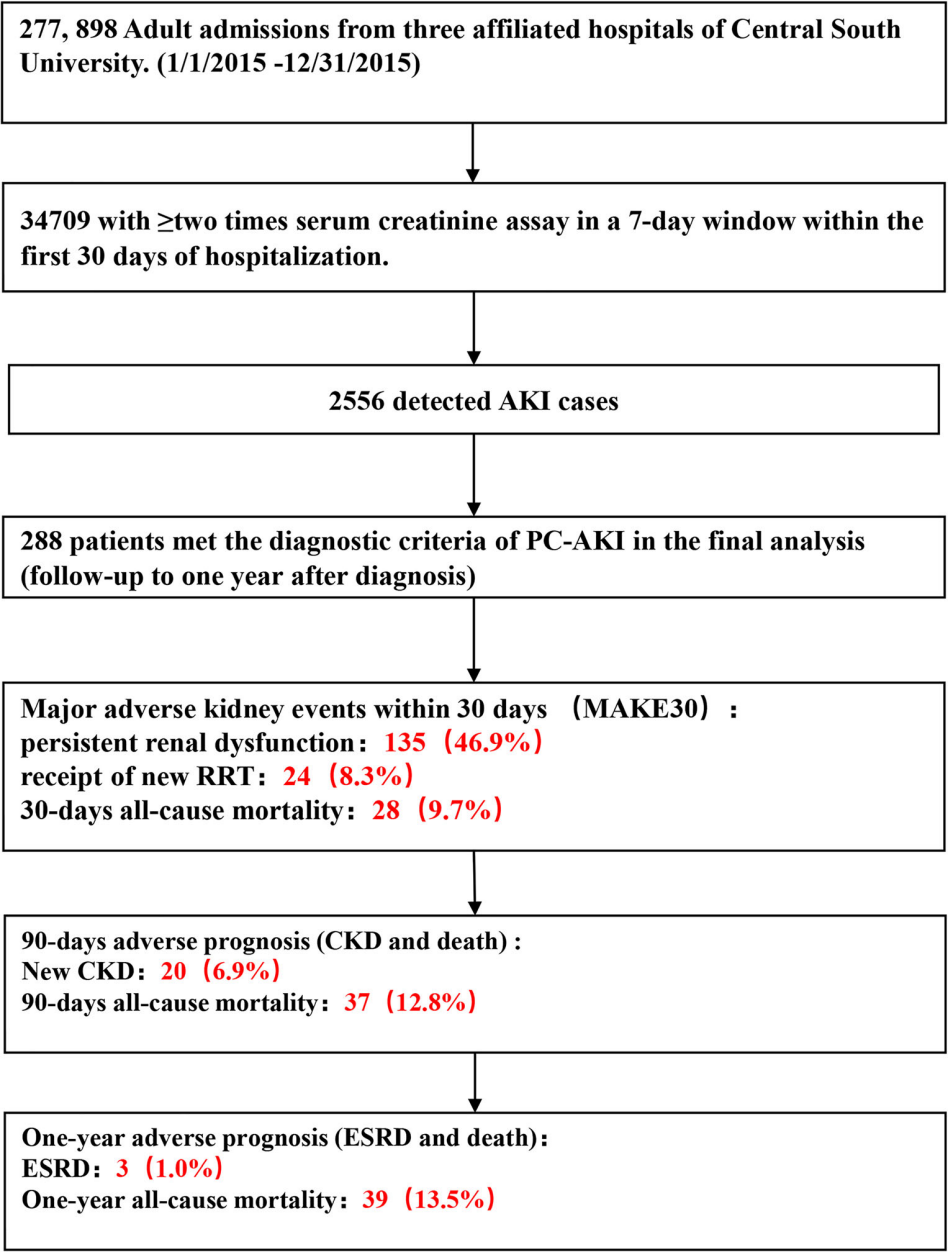
Study flow diagram

Our multi-center population-based cohort study that was conducted in three affiliated hospitals of Central South University in China between January 1 and December 31, 2015, included 277,898 adult hospitalized patients (more than 18 years old). We selected patients from the cohort who had at least two SCr tests within any 7-day window during their first 30 days of hospitalization and diagnosed as the PC-AKI, meeting the PC-AKI diagnostic criteria of 2018 guidelines of the ESUR [3]. Patients with ESRD or requiring RRT, with hospital stay < 48 h or incomplete medical records, with follow-up < 1 year or loss were excluded. Patients who met one of the following criteria were excluded: use of nephrotoxic drugs (like gentamicin, acyclovir, or cisplatin); suffering from malignant tumors; hepatorenal syndrome; suffering from autoimmune disease; pregnancy. For patients with multiple hospitalizations, we included only the first hospitalization in the analysis set. All participants were followed up for 1 year from admission. This retrospective observational study was approved by the Medical Ethics Committee of the Second Xiangya Hospital of Central South University (approval number 2013-S061) and the need for informed consent was waived, considering the retrospective study design (Fig. 1).

### Definition of start point and endpoint

PC-AKI is defined as an increase in SCr ≥ 0.3 mg/dL (≥ 26.4 μmol/L) or ≥ 1.5 times baseline within 48–72 h of intravascular administration of a contrast medium according to the 2018 guidelines of ESUR [3]. Time of the baseline SCr value measurement was within 72 h before contrast medium administration. Persistent RD was defined as final serum creatinine value before hospital discharge ≥ 200% of the baseline SCr value in a patient not known to have previously received RRT; MAKE30 was defined as in-hospital mortality, receipt of new RRT, or persistent RD [20, 21]; CKD was defined as abnormalities of kidney function or structure, or a sustained outpatient estimated glomerular filtration rate (eGFR) value of less than 60 mL/min/1.73 m^2^ for more than 3 months according to the modified glomerular filtration rate estimating (MDRD) equation [22, 23]. ESRD was defined as eGFR < 15 mL/min/1.73m^2^ or receipt of any form of RRT [24]. The primary endpoint of the observation was 30-day, 90-day, and 1-year all-cause mortality. The terminal point of the observation was the proportion of patients who met one or more criteria for MAKE30, incidence of CKD within 90 days, and incidence of ESRD within 1 year.

### Definition of data sources

We obtained patient-level data from the electronic hospitalization databases and laboratory databases from the participating hospitals. The hospitalization records consisted of patients’ age; sex; X-ray procedures, including computed tomography (CT), computed tomography angiography (CTA), and percutaneous coronary interventions (PCI); type of contrast media including iodixanol, iohexol, iopromide, iopamidol, and ioversol (more than one type of contrast media); volumes of injected contrast media; and oliguria or anuria. Medical history includes hypertension; diabetes; CKD or cardiopulmonary bypass surgery; and complications of AKI including hyperkalemia, metabolic acidosis, heart failure, respiratory failure, shock, central nervous system failure, and gastrointestinal bleeding [25]. The laboratory data included red cell volume distribution width (RDW) [26], hemoglobin (Hb), platelet (PLT), serum albumin (Alb), blood urea nitrogen (BUN), total cholesterol (TC), baseline eGFR (we estimated GFR according to the MDRD equation [23] and expressed relative GFR in mL/min/1.73 m^2^ recommended by ESUR [27]), total bilirubin (TBIL) [28], proteinuria, and AKI stage [29]. Drugs used within 48 h prior to or after exposure to contrast media included diuretics and nephroprotective drugs (*N*-acetylcysteine or sodium bicarbonate).

### Statistical analysis

The collected data were used to establish a qualified database and statistically analyzed by using SPSS 18.0. The data of normal distribution were presented with mean ± standard deviation (mean ± SD). The continuous variables were expressed by mean and standard deviations and compared by *t* tests. The enumeration data were expressed by rate and compared by chi-square test. Kaplan-Meier, multivariable Cox regression, and logistic regression (forward stepwise selection) analyses were used to analyze main outcomes and risk factors after adjusted candidate variables by log-rank test. The criterion for selecting the adjusted candidate variables is *α*_enter_ = 0.05, CI = 95%. The criterion for excluding the adjusted candidate variables is *α*_remove_ = 0.10, CI = 95%. *p* value less than 0.05 was considered to be statistically significant.

## Results

### Clinical characteristics

Table 1 presents the baseline characteristics of PC-AKI patients. The overall incidence of PC-AKI in hospitalized patients was 0.83% (288/34709) including 180 males and 108 females. X-ray procedures the PC-AKI patients underwent were available, including CT (52.4%), CTA (33.0%), and PCI (14.6%). The types of contrast media included the following: iohexol (92, 31.9%), iopromide (67, 23.3%), iopamidol (60, 31.9%), or ioversol (43, 14.9%), and iodixanol (12, 4.2%), and others (more than one type; 14, 4.9%). In addition, the injected contrast volume was less than 50 mL in 21 patients (7.3%) and at least 100 mL in 111 patients (38.5%). For the remaining 156 patients (54.2%), the volume of injected contrast media ranged from 50 to 100 mL. When stratifying complications of PC-AKI, we found that the top one was heart failure. The proportion of basic eGFR in PC-AKI patients was 88.9% (≥ 60 mL/min/1.73m^2^), 8.0% (45–60 mL/min/1.73m^2^), 2.8% (30–45 mL/min/1.73m^2^), and 0.3% (15–30 mL/min/1.73m^2^), respectively. All 145 (50.3%) patients did not stop diuretics and only 30 (10.4%) patients used nephroprotective drugs. Table S1 presents the baseline characteristics of persistent RD and transient RD patients.

**Table 1:** The clinical, laboratory basic data of PC-AKI patients

Characteristic	Cohort, no. (%) of PC-AKI patients
Clinical data	
Age, years	55.61 ± 13.26
Age ≥ 65 years, no. (%)	76 (26.4)
Gender (women), no. (%)	108 (37.5)
Oliguria or anuria^a^, no. (%)	34 (11.8)
X-ray procedures, no. (%)	
CT	151 (52.4)
CTA	95 (33.0)
PCI	42 (14.6)
Type of contrast media, no. (%)	
Iodixanol	12 (4.2)
Iohexol	92 (31.9)
Iopromide	67 (23.3)
Iopamidol	60 (20.8)
Ioversol	43 (14.9)
Others	14 (4.9)
Volumes of injected contrast media, mL	86.01 ± 43.91
< 50 mL, no. (%)	21 (7.3)
≥ 50 mL, < 100 mL, no. (%)	156 (54.2)
≥ 100 mL, no. (%)	111 (38.5)
Complications of AKI^b^, no. (%)	
Hyperkalemia	19 (6.6)
Metabolic acidosis	60 (20.8)
Heart failure	75 (26.0)
Respiratory failure	34 (11.8)
Shock	35 (12.2)
Central nervous system failure	46 (16.0)
Gastrointestinal bleeding	16 (5.6)
Medical history, no. (%)	
Hypertension	115 (39.9)
Diabetes	51 (17.7)
Chronic kidney disease	42 (14.6)
Cardiopulmonary bypass surgery	59 (20.5)
Laboratory data^c^	
Hemoglobin (Hb), g/L	110.06 ± 26.18
Anemia (Hb < 100 g/L), no. (%)	74 (25.7)
Alb, g/L	34.92 ± 6.63
Hypoalbuminemia (Alb < 30 g/L), no. (%)	53 (18.6)
Total cholesterol (TC), mmol/L	4.16 ± 1.70
Hyperlipidemia (TC ≥ 6.22 mmol/L), no. (%)	16 (5.6)
Total bilirubin, μmol/L	32.75 ± 67.71
Total bilirubin, no. (%)	
0 < 20 μmol/L	191 (67.0)
20–32 μmol/L	51 (17.9)
33–101 μmol/L	22 (7.7)
102–204 μmol/L	12 (4.2)
> 204 μmol/L	9 (3.2)
Blood urea nitrogen, mmol/L	11.66 ± 8.20
Blood urea nitrogen ≥ 7.14 mmol/L, no. (%)	182 (63.2)
RDW-CV, (%)	14.23 ± 2.64
RDW-CV ≥ 13.7%, no. (%)	146 (50.7)
PLT, 10^9^/L	169.66 ± 95.04
PLT < 100 or > 300 × 10^9^/L	91 (31.6)
The baseline eGFR (mL/min/1.73 m^2^)	88.35 ± 23.61
The baseline eGFR^d^, no. (%)	
15–30 mL/min/1.73 m^2^	1 (0.3)
30–45 mL/min/1.73 m^2^	8 (2.8)
45–60 mL/min/1.73 m^2^	23 (8.0)
≥ 60 mL/min/1.73 m^2^	256 (88.9)
Proteinuria^e^, no. (%)	66 (22.9)
Acute kidney injury stage^f^, no. (%)	
Stage 1	158 (54.9)
Stage 2	69 (24.0)
Stage 3	61 (21.2)
Use of diuretics	145 (50.3)
Use of nephroprotective drugs	30 (10.4)

^a^Oliguria or anuria (urine volume < 400 or 100 mL/24 h)

^b^Complications of AKI: hyperkalemia (serum K^+^ peak value > 5.5 mmol/L), metabolic acidosis (an arterial blood pH 7.35 with plasma bicarbonate 22 mmol/L), heart failure (based on Framingham criteria and defined as New York Heart Association functional class IV), respiratory failure (need for mechanical ventilation), shock (hypotension with systolic arterial blood pressure lower than 90 mmHg despite adequate fluid resuscitation), central nervous system failure (progressive coma), gastrointestinal bleeding (upper gastrointestinal bleeding and lower gastrointestinal bleeding)

^c^The worst value was taken within 7 days

^d^The estimated GFR according to modified glomerular filtration rate estimating equation

^e^Proteinuria as dipstick urinalysis protein positive

^f^According to three categories of KDIGO staging system based on the highest SCr value identified during hospitalization

### PC-AKI and MAKE30

**Fig. 2:**
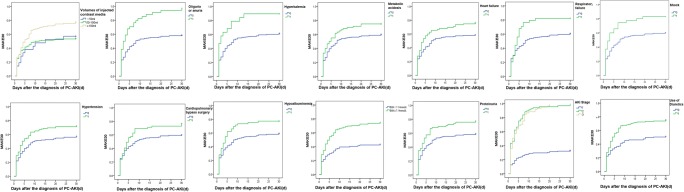
Kaplan-Meier analysis of risk factors for MAKE30 in patients with PC-AKI (0 = none, 1 = yes, *p* < 0.05)

Of the 288 PC-AKI patients, 153 PC-AKI patients (53.1%) had experienced transient RD and had returned to baseline (or near baseline); 135 of these (46.9%) presented persistent RD. The 30-day all-cause mortality rate was 9.7% (28/288) and the incidence of new receipt of RRT within 30 days was 8.3% (24/288) (Table 2). The results showed 14 variables including volumes of injected contrast media, oliguria or no urine, hyperkalemia, acidosis, heart failure, respiratory failure, hypotension shock, hypertension or cardiopulmonary bypass surgery, hypoalbuminemia, BUN ≥ 7.14 mmol/L, proteinuria, AKI stage, and diuretic injection were associated with higher incidence of MAKE30 (log rank method, *p* < 0.05) (Fig. 2; Table S2). The incidence of MAKE30 of patients who received contrast media volumes more than 100 mL (≥ 100 mL) was significantly higher than that of those who received contrast volumes below 100 mL (log rank method, *p* < 0.05). However, the type of contrast media injected, as well as use of nephroprotective drugs, was not associated significantly with the overall incidence of MAKE30 (Figs. S1-S2; Table S2). Multivariable Cox regression analysis showed KDIGO AKI stage was independently associated with MAKE30 after adjusted for the fourteen variables above (*p* < 0.001). Worsening AKI stage was correlated with increased risk of MAKE30, and the risk ratio (RR) for AKI stage 2 or 3 (versus AKI stage 1) was 7.027 (95% CI, 4.918–10.039).

**Table 2:** The adverse prognosis in patients with PC-AKI

Adverse prognosis of PC-AKI patients	Cohort, no. (%) of PC-AKI patients
Major adverse kidney events within 30 days	
Persistent renal dysfunction (serum creatinine value ≥ 200% the baseline serum creatinine value)	135 (46.9)
Receipt of new RRT	24 (8.3)
All-cause mortality	28 (9.7)
90-day adverse prognosis (CKD and death)	
New CKD	20 (6.9)
90-day all-cause mortality	37 (12.8)
1-year adverse prognosis	
ESRD	3 (1.0)
1-year all-cause mortality	39 (13.5)

*RRT*, renal replacement therapy; *CKD*, chronic kidney disease; *ESRD*, end-stage renal disease

### PC-AKI and 90-day clinical outcome

**Fig. 3:**
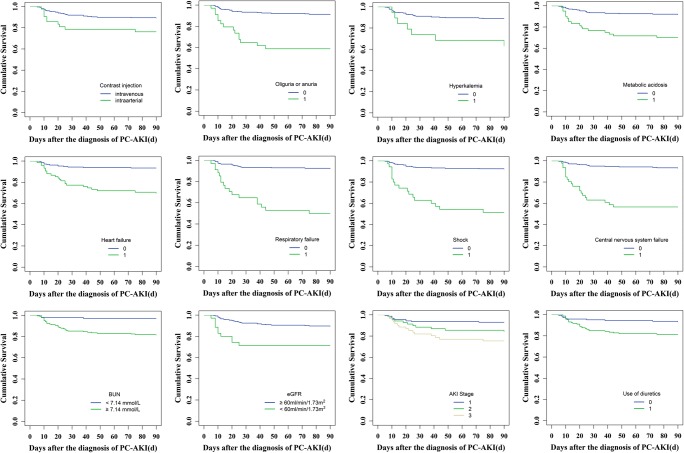
Kaplan-Meier analysis of risk factors for 90-day all-cause mortality in patients with PC-AKI (0 = none, 1 = yes, *p* < 0.05)

The incidence of new CKD within 90 days after PC-AKI was 6.9% (20/288) and the 90-day all-cause mortality rate was 13.5% (39/288) (Table 2). Contrast injection (intravenous and intra-arterial), oliguria or anuria, hyperkalemia, acidosis, heart failure, respiratory failure, hypotension shock, central nervous system failure, BUN ≥ 7.14 mmol/L, AKI stages, baseline eGFR < 60 mL/min/1.73 m^2^, and diuretic injection, as well as persistent renal dysfunction and dialysis within 30 days, were associated with 90-day all-cause mortality (log rank method, *p* < 0.05) (Figs. 3 and 5; Table S3). The 90-day all-cause mortality of PC-AKI patients who underwent intra-arterial procedure (PCI) was much higher than that of those who underwent intravenous CT procedures (enhanced CT and CTA) (log rank method, *p* < 0.05). Patients with severe AKI (AKI stage ≥ 2) had higher 90-day all-cause mortality rate (log rank method, *p* < 0.001). However, types or volume of contrast media, as well as use of nephroprotective drugs, was not significantly associated with 90-day all-cause mortality (Figs. S1-S2; Table S3). Multivariable Cox regression and logistic regression analyses showed persistent RD in 30 days, heart failure, central nervous system failure, and baseline eGFR < 60 mL/min/1.73 m^2^ were independent risk factors for 90-day all-cause mortality or combined clinical endpoints (CKD and 90-day all-cause mortality) (*p* < 0.05). Cox proportional hazard regression analysis showed that PC-AKI patients with persistent RD had a higher all-cause mortality rate of 90 days than those with transient RD (RR = 3.768; 95% CI, 1.612–8.810; *p* = 0.002) (Table 3). According to the multivariable logistic regression analysis, the risk of death was higher in PC-AKI patients with persistent RD than that in patients with transient RD (odds ratio, OR = 3.685; 95% CI, 1.628–8.340; *p* = 0.002). In addition, BUN ≥ 7.14 mmol/L and oliguria or anuria were also identified as independent predictors for combined clinical endpoints (*p* < 0.05) (Table 3).

**Table 3:** Risk factors for 90-day all-cause mortality (multivariable Cox regression survival analysis) and 90-day adverse outcomes (CKD and mortality) (multivariable logistic regression analysis) in patients with PC-AKI

Variables	Multivariable Cox regression survival analysis		Multivariable logistic regression analysis	
	RR value (95% CI)	*p* value	OR value (95% CI)	*p* value
Persistent renal dysfunction^a^	3.768 (1.612–8.810)	0.002	3.685 (1.628–8.340)	0.002
Heart failure	2.433 (1.149–5.153)	0.020	2.655 (1.267–5.566)	0.010
Central nervous system failure	4.830 (2.303–10.130)	0.000	6.640 (2.857–15.433)	0.000
The baseline eGFR < 60 mL/min/1.73 m^2^	2.665 (1.259–5.642)	0.010	2.739 (1.095–6.851)	0.031
BUN ≥ 7.14 mmol/L			2.933 (1.087–7.913)	0.034
Oliguria or anuria			3.275 (1.299–8.255)	0.012

^a^Serum creatinine value ≥ 200% the baseline serum creatinine value

*BUN*, blood urea nitrogen

Adjusted variables: contrast injection, oliguria or anuria, hyperkalemia, acidosis, heart failure, respiratory failure, hypotension shock, central nervous system failure, BUN ≥ 7.14 mmol/L, baseline eGFR < 60 mL/min/1.73 m^2^, diuretics injected, persistent renal dysfunction, and dialysis within 30 days

### PC-AKI and 1-year clinical outcome

**Fig. 4:**
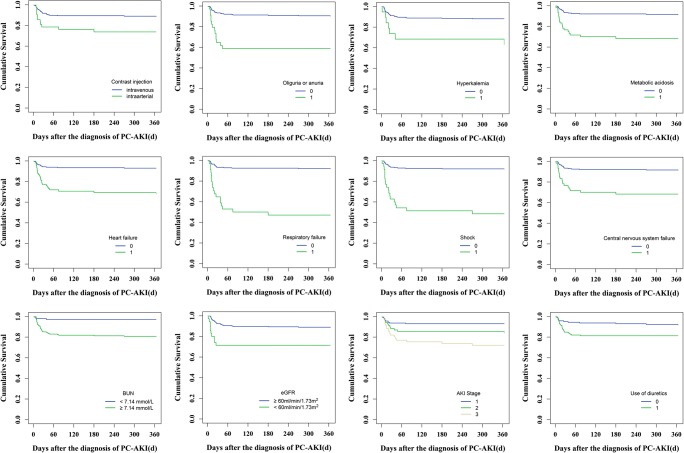
Kaplan-Meier analysis of risk factors for 1-year all-cause mortality in patients with PC-AKI (0 = none, 1 = yes, *p* < 0.05)

**Fig. 5:**
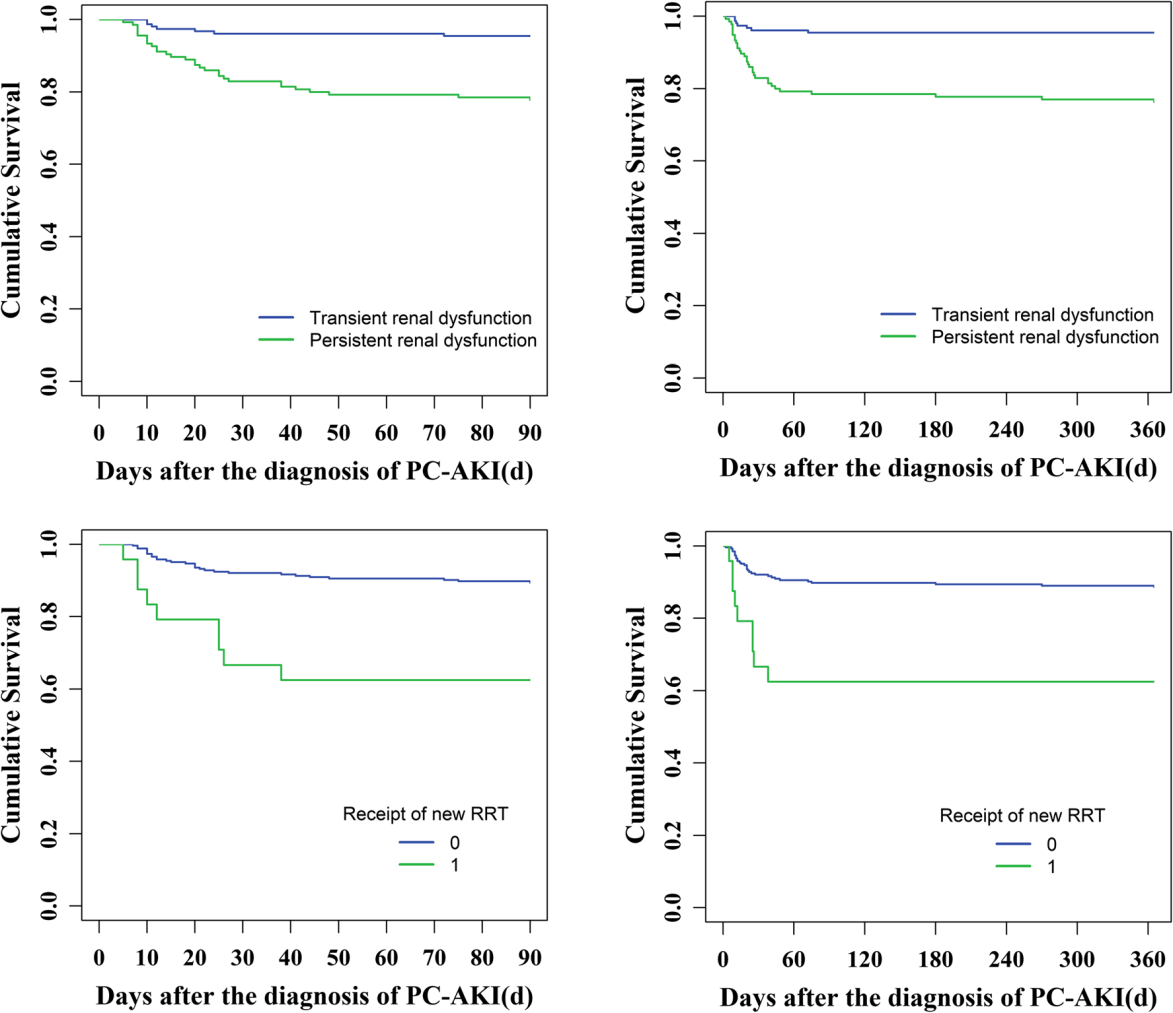
Prognosis of PC-AKI patients with or without persistent RD or RRT (0 = none, 1 = yes, *p* < 0.05)

The incidence of ESRD within 1 year was 1.0% (3/288); the 1-year all-cause mortality rate was 13.5% (39/288) (Table 2). In Kaplan-Meier analysis, fourteen variables were associated with 1-year all-cause mortality, which were consistent with the risk factors for 90-day all-cause mortality (Figs. 4 and 5; Table S4). The 1-year all-cause mortality rate of PC-AKI patients who underwent intra-arterial procedure (PCI) was much higher than that of those who underwent intravenous CT procedures (log rank method, *p* < 0.05). The 1-year all-cause mortality rate got higher with the increase of AKI stage (AKI stage ≥ 2) (log rank method, *p* < 0.001), but not associated significantly with types or volume of contrast media (Fig. S1; Table S4). Besides, nephroprotective drugs injected prior to, after, or both prior to and after exposure to contrast media did not improve long-term prognosis of PC-AKI patients (Fig. S2; Table S5). We adopted multivariable Cox regression and logistic regression analyses to calculate the adjusted RR or OR value and 95% CIs for the 1-year clinical outcome. In the multivariable Cox regression analysis of 288 PC-AKI patients who were followed up for 1 year, persistent RD was proved to be an independent predictor of 1-year outcome. As shown in Table 4, the risk of 1-year all-cause mortality in persistent RD patients increased by 4.106-fold and the risk of combined clinical endpoints (ESRD and 1-year all-cause mortality) increased by 5.209-fold compared with that in transient RD patients (RR = 4.106, 95% CI 1.765–9.551, *p* = 0.001; OR = 5.209, 95% CI 1.730–15.681, *p* = 0.003). Additional covariates, identified as independent predictors of 1-year all-cause mortality or combined clinical endpoints in multivariable Cox regression and logistic regression analyses, were heart failure, central nervous system failure, baseline eGFR < 60 mL/min/1.73 m^2^, and respiratory failure (*p* < 0.05). In addition, hypotension shock was independently associated with combined clinical endpoints (ESRD and 1-year all-cause mortality) (OR = 3.367, 95% CI 1.084–10.454, *p* = 0.036) (Table 4).

**Table 4:** Risk factors for 1-year all-cause mortality (multivariable Cox regression survival analysis) and 1-year adverse outcomes (ESRD and mortality) (multivariable logistic regression analysis) in patients with PC-AKI

Variables	Multivariable Cox regression survival analysis		Multivariable logistic regression analysis	
	RR value (95% CI)	*p* value	OR value (95% CI)	*p* value
Persistent renal dysfunction^a^	4.106 (1.765–9.551)	0.001	5.209 (1.730–15.681)	0.003
Heart failure	2.366 (1.141–4.907)	0.021	3.402 (1.356–8.538)	0.006
Central nervous system failure	4.981 (2.423–10.240)	0.000	7.685 (2.633–22.430)	0.000
The baseline eGFR < 60 mL/min/1.73 m^2^	2.567 (1.223–5.424)	0.013	3.528 (1.186–10.496)	0.023
Respiratory failure	2.281(1.046–4.976)	0.038	3.895 (1.403–10.808)	0.009
Shock			3.367 (1.084–10.454)	0.036

^a^Serum creatinine value ≥ 200% the baseline serum creatinine value

Adjusted variables: contrast injection, oliguria or anuria, hyperkalemia, acidosis, heart failure, respiratory failure, hypotension shock, central nervous system failure, BUN ≥ 7.14 mmol/L, baseline eGFR < 60 mL/min/1.73 m^2^, diuretics injected, persistent renal dysfunction, and dialysis within 30 days

## Discussion

The results of our observational study suggest that the incidence of PC-AKI in a hospitalized population was about 0.83% (288/34709). PC-AKI can result in adverse outcome such as persistent RD, dialysis, CKD, ESRD, or mortality. PC-AKI patients with severe AKI (stage ≥ 2) had a more frequent incidence of MAKE30. PC-AKI patients with persistent RD, heart failure, central nervous system failure, baseline eGFR < 60 mL/min/1.73 m^2^, oliguria or anuria, blood urea nitrogen ≥ 7.14 mmol/L, respiratory failure, and hypotension shock were prone to long-term adverse prognosis.

PC-AKI is a severe complication of intravascular applied radiological contrast media. Previous research showed CI-AKI accounts for 11% of the causes of AKI [30]. Jin et al [31] reported that 45.9% of CI-AKI patients who received PCI developed persistent renal dysfunction within 30 days (defined as an increase by 25% or 0.5 mg/dL or 44.2 μmol/L in SCr level relative to the baseline). Maioli et al [32] showed that in a follow-up study of 180 patients with CI-AKI, 31 patients (18.6%) presented persistent renal dysfunction (defined as a relative 25% decrease of creatinine clearance over baseline for 3 months). James et al reported that 2.4% (18/742) of mild PC-AKI and 5.8% (8/137) of moderate or severe PC-AKI initiated chronic RRT within 2 years after the diagnosis [33]. Another study showed that 1.6% of patients with PC-AKI stage 1 and 11.5% of patients with PC-AKI stage 2/3 developed ESRD after a median follow-up of 19.7 months [34]. Our study indicated that the incidence of PC-AKI in hospitalized population was 0.83% and that about 46.9% of the patients developed persistent RD and 8.3% needed new RRT within 30 days thereafter. New-onset CKD occurred in 20 (6.9%) of 288 patients with PC-AKI after 3 months and 1.0% patients finally depended on dialysis permanently within 1 year. In addition, our results also showed that, in three affiliated hospitals of Central South University in China, the all-cause mortality of PC-AKI patients within 30 days, 90 days, and 1 year was 9.7% (28/288), 12.8% (37/288), and 13.5% (39/288), respectively. Charanjit et al [13] found that the mortality rates of patients experiencing PC-AKI after PCI at 6 months, 1 year, and 5 years were 9.8%, 12.1%, and 44.6%, respectively. James et al [33] pointed out that mortality rate was 16.0% in PC-AKI patients in 3 years. The discrepancy in incidence of adverse outcome and mortality of patients with CI-AKI between each study may be related to the inclusive and exclusive criteria of patients, and endpoint time of observations. In summary, the above results indicate that PC-AKI is not always a transient, benign creatininopathy, but rather a direct cause of worsening renal function, resulting in adverse outcome such as persistent renal dysfunction, even dialysis, and mortality.

MAKE is increasingly being recommended [16] and used [19] as the endpoint of choice for AKI clinical trials [15, 18, 35–38]. Two kidney-specific events (receipt of new RRT and persistent RD) may be more closely related to long-term morbidity and quality of life than transient elevations in creatinine [39, 40]. Our study indicated that the association between PC-AKI and MAKE may involve several mechanisms. First, complications or clinical features, oliguria or anuria, hyperkalemia, acidosis, heart failure, respiratory failure, hypotension shock, central nervous system failure, BUN ≥ 7.14 mmol/L, hypoalbuminemia, or proteinuria can increase the risk of MAKE30. Second, underlying disease like hypertension or cardiopulmonary bypass surgery also affected the short-term adverse prognosis. However, there was no significant difference in the incidence of MAKE30 regardless of the patient’s previous history of diabetes or CKD. Third, in short-term, renal function is considered to be affected by the large amount of contrast volume, but it is not related to the type and injection route of the contrast media. Fourth, there was an exacerbation of renal dysfunction when diuretics were injected and nephroprotective drugs did not improve short-term prognosis of PC-AKI patients. Furthermore, our study proved that the AKI stage was independently correlated with MAKE30 and severe AKI (stage ≥ 2) had more frequent incidence of MAKE30. Similarly, James et al reported that the proportion of CI-AKI patients with persistent RD (defined as an increase of serum creatinine concentration by 50% or 0.3 mg/dL from baseline, maintaining more than 3 months) was 5.9% in patients without AKI, 28.2% in patients with mild AKI, and up to 59.1% in patients with moderate or severe AKI [33].

In most cases, PC-AKI was transient, and renal function recovered almost completely within 3 months [41, 42]. However, PC-AKI patients with heart failure and central nervous system failure indicated higher mortality or renal dysfunction (CKD, ESRD). Blood urea nitrogen ≥ 7.14 mmol/L or oliguria or anuria suggested a higher risk of progression to CKD, while PC-AKI patients with shock and respiratory failure were prone to death or ESRD within 1 year. It indicated that long-term adverse outcome of PC-AKI patients was substantially influenced by baseline clinical features, especially those that predispose to both kidney injury and mortality. This was consistent with another meta-analysis [8]. Pre-existing severe renal insufficiency has been proposed as an important risk factor for CI-AKI [4, 43–45]. In our study, the baseline eGFR < 60 mL/min/1.73 m^2^ was also the independent risk factor for progression to CKD or ESRD or all-cause mortality in PC-AKI patients. Therefore, it is necessary to adopt a policy of SCr measurements [46] in all patients scheduled for iodine-based contrast media injection. Renal protective drugs and preventive hydration can be used to reduce the incidence of PC-AKI in at-risk patients [4, 47], but our results showed that it did not improve the long-term mortality of PC-AKI patients. In addition, dialysis as RRT to remove ICM [48] has not been shown to be beneficial on the prognosis of PC-AKI, but there was an exacerbation of renal dysfunction and higher long-term mortality when diuretics were used. Stopping diuretics 48 h before the examination can limit ICM-induced renal toxicity [2] and effectively improves PC-AKI prognosis. Several studies have failed to establish a clear advantage of IV CM over IA CM [49–52] and iso-osmolar contrast media (IOCM) over low-osmolar contrast media (LOCM) [53–57], with regard to incidence of PC-AKI or CI-AKI. Castaldo P et al [58] confirmed that IV CM administration carries a low incidence of renal impairment. Moreover, there is insufficient evidence that CM dose is a risk factor when CM is injected intravenously [3]. Our study indicated that long-term mortality of PC-AKI patients who underwent intra-arterial procedure (PCI) was much higher than that of those who underwent intravenous CT procedures, but there is no significant correlation with the type of contrast media and the injected volume. It is noticeable that the effects of route of CM administration on adverse outcome should not be separated from the effects of surgical manipulations, co-morbidity, or other baseline clinical characteristics. For instance, Sohn KH et al [59] showed that previous exposure to ICM via intra-arterial route was a significant risk factor for immediate hypersensitivity to IA CM. Besides, it may be partly related to the hospital policies on strict control of the type and volumes of contrast media used: iodixanol was recommended in patients at risk, while LOCM is more diverse and more widely used in the clinical practice, and it is advisable to keep the volume of ICM administered as low as possible.

Multivariable Cox and logistic regression analyses showed that persistent RD occurring within 30 days was the independent risk factor for progression to CKD or ESRD or all-cause mortality, which suggested that the further reduction in glomerular filtration rate might amplify the risk of long-term adverse events. Compared with PC-AKI patients with transient renal damage, the risk of 90-day or 1-year all-cause mortality was 3.8 times or 4.1 times higher in persistent RD patients. What is more, persistent RD patients had 3.7-fold or 5.2-fold increase in risk of 90-day or 1-year adverse prognosis versus PC-AKI transient RD, respectively. Similarly, Jin et al [31] reported that PC-AKI patients with persistent RD (impaired renal function within 1 month) after PCI had a higher 2-year mortality rate (34.6% vs. 16.7%), a higher death or dialysis rate (34.1% vs. 17.9%), and a higher death, dialysis, or hospital admission rate due to cardiovascular events (42.1% vs. 22.9%) than those with transient RD. Persistent RD was an important intermediate stage in progression to the long-term adverse prognosis after PC-AKI, which can effectively predict the poor prognosis of patients with PC-AKI. So early intervention for the risk factors of persistent RD may effectively reduce the incidence of MAKE30 and even improve the long-term prognosis of patients with PC-AKI.

Our study has several limitations. The main limitation of the present study is the relatively small sample size. In fact, the low rate of PC-AKI implies the need for a large population to start. Besides, it was a follow-up observation study, so multi-center prospective trials are still necessary to consider prevention of PC-AKI.

In conclusion, PC-AKI is not always a transient, benign creatininopathy, but can result in adverse outcome. AKI stage is independently correlated to MAKE30 and persistent renal dysfunction may exaggerate the risk of long-term adverse events.

## Electronic supplementary material

ESM 1 (DOCX 455 kb)

ESM 2 (TIF 1.44 MB)

ESM 3 (TIF 1.72 MB)

## Abbreviations

ACR: American College of Radiology
Alb: Albumin
BUN: Blood urea nitrogen
CI-AKI: Contrast-induced acute kidney injury
CKD: Chronic kidney disease
CMSC: Contrast Media Safety Committee
CT: Computed tomography
ERBP: European Renal Best Practice
ESRD: Even end-stage renal disease
ESUR: European Society of Urogenital Radiology
Hb: Hemoglobin
IA CM: Intra-arterial contrast media
ICM: Iodine-based contrast media
IV CM: Intravenous contrast media
KDIGO: Kidney Disease: Improving Global Outcomes
MAKE: Major adverse kidney events
MDRD: Modified glomerular filtration rate estimating
OR: Odds ratio
PCI: Percutaneous coronary interventions
RDW: Red cell volume distribution width
RR: Risk ratio
RRT: Renal replacement therapy
SCr: Serum creatinine
TBIL: Total bilirubin
TC: Total cholesterol

## References

[1] Rear R, Bell RM, Hausenloy DJ (2016). Contrast-induced nephropathy following angiography and cardiac interventions. Heart.

[2] Faucon AL, Bobrie G, Clement O (2019). Nephrotoxicity of iodinated contrast media: from pathophysiology to prevention strategies. Eur J Radiol.

[3] van der Molen AJ, Reimer P, Dekkers IA (2018). Post-contrast acute kidney injury - part 1: definition, clinical features, incidence, role of contrast medium and risk factors: recommendations for updated ESUR Contrast Medium Safety Committee guidelines. Eur Radiol.

[4] ACR Committee on Drugs and Contrast Media (2017) ACR Manual on Contrast Media, v10.3. American College of Radiology. Available via: https://www.acr.org/-/media/ACR/Files/ClinicalResources/Contrast_Media.pdf Accessed: 14 December 2017

[5] Ad-hoc working group of ERBP, Fliser D, Laville M et al (2012) A European Renal Best Practice (ERBP) position statement on the Kidney Disease Improving Global Outcomes (KDIGO) clinical practice guidelines on acute kidney injury: part 1: definitions, conservative management and contrast-induced nephropathy. Nephrol Dial Transplant 27:4263–4272 10.1093/ndt/gfs375 PMC3520085 23045432

[6] Tao SM, Wichmann JL, Schoepf UJ, Fuller SR, Lu GM, Zhang LJ (2016). Contrast-induced nephropathy in CT: incidence, risk factors and strategies for prevention. Eur Radiol.

[7] Brar SS (2018). Protocol-driven CI-AKI prevention in the Cath lab. J Am Coll Cardiol.

[8] James MT, Samuel SM, Manning MA (2013). Contrast-induced acute kidney injury and risk of adverse clinical outcomes after coronary angiography: a systematic review and meta-analysis. Circ Cardiovasc Interv.

[9] Kooiman J, Seth M, Nallamothu BK, Heung M, Humes D, Gurm HS (2015). Association between acute kidney injury and in-hospital mortality in patients undergoing percutaneous coronary interventions. Circ Cardiovasc Interv.

[10] Mitchell AM, Kline JA, Jones AE, Tumlin JA (2015). Major adverse events one year after acute kidney injury after contrast-enhanced computed tomography. Ann Emerg Med.

[11] Rudnick M, Feldman H (2008). Contrast-induced nephropathy: what are the true clinical consequences?. Clin J Am Soc Nephrol.

[12] Gupta R, Gurm HS, Bhatt DL, Chew DP, Ellis SG (2005). Renal failure after percutaneous coronary intervention is associated with high mortality. Catheter Cardiovasc Interv.

[13] Rihal CS, Textor SC, Grill DE (2002). Incidence and prognostic importance of acute renal failure after percutaneous coronary intervention. Circulation.

[14] Gruberg L, Mintz GS, Mehran R (2000). The prognostic implications of further renal function deterioration within 48 h of interventional coronary procedures in patients with pre-existent chronic renal insufficiency. J Am Coll Cardiol.

[15] Kashani K, Al-Khafaji A, Ardiles T (2013). Discovery and validation of cell cycle arrest biomarkers in human acute kidney injury. Crit Care.

[16] Palevsky PM, Molitoris BA, Okusa MD (2012). Design of clinical trials in acute kidney injury: report from an NIDDK workshop on trial methodology. Clin J Am Soc Nephrol.

[17] Kellum John A, Lameire Norbert (2013). Diagnosis, evaluation, and management of acute kidney injury: a KDIGO summary (Part 1). Critical Care.

[18] Shaw A (2011). Models of preventable disease: contrast-induced nephropathy and cardiac surgery-associated acute kidney injury. Contrib Nephrol.

[19] Weisbord SD, Gallagher M, Kaufman J (2013). Prevention of contrast-induced AKI: a review of published trials and the design of the prevention of serious adverse events following angiography (PRESERVE) trial. Clin J Am Soc Nephrol.

[20] Semler MW, Rice TW, Shaw AD (2016). Identification of major adverse kidney events within the electronic health record. J Med Syst.

[21] Kellum JA, Zarbock A, Nadim MK (2017). What endpoints should be used for clinical studies in acute kidney injury?. Intensive Care Med.

[22] Luo M, Yang Y, Xu J (2017). A new scoring model for the prediction of mortality in patients with acute kidney injury. Sci Rep.

[23] Ma YC, Zuo L, Chen JH (2006). Modified glomerular filtration rate estimating equation for Chinese patients with chronic kidney disease. J Am Soc Nephrol.

[24] Andrassy KM (2013). Comments on ‘KDIGO 2012 clinical practice guideline for the evaluation and management of chronic kidney disease’. Kidney Int.

[25] Marshall JC, Cook DJ, Christou NV, Bernard GR, Sprung CL, Sibbald WJ (1995). Multiple organ dysfunction score: a reliable descriptor of a complex clinical outcome. Crit Care Med.

[26] Xiong XF, Yang Y, Chen X (2017). Red cell distribution width as a significant indicator of medication and prognosis in type 2 diabetic patients. Sci Rep.

[27] Nyman U, Ahlkvist J, Aspelin P (2018). Preventing contrast medium-induced acute kidney injury : side-by-side comparison of Swedish-ESUR guidelines. Eur Radiol.

[28] Zheng YX, Zhong X, Li YJ, Fan XG (2017). Performance of scoring systems to predict mortality of patients with acute-on-chronic liver failure: a systematic review and meta-analysis. J Gastroenterol Hepatol.

[29] Khwaja A (2012). KDIGO clinical practice guidelines for acute kidney injury. Nephron Clin Pract.

[30] Nash K, Hafeez A, Hou S (2002). Hospital-acquired renal insufficiency. Am J Kidney Dis.

[31] Wi J, Ko YG, Kim JS (2011). Impact of contrast-induced acute kidney injury with transient or persistent renal dysfunction on long-term outcomes of patients with acute myocardial infarction undergoing percutaneous coronary intervention. Heart.

[32] Maioli M, Toso A, Leoncini M, Gallopin M, Musilli N, Bellandi F (2012). Persistent renal damage after contrast-induced acute kidney injury: incidence, evolution, risk factors, and prognosis. Circulation.

[33] James MT, Ghali WA, Tonelli M (2010). Acute kidney injury following coronary angiography is associated with a long-term decline in kidney function. Kidney Int.

[34] James MT, Ghali WA, Knudtson ML (2011). Associations between acute kidney injury and cardiovascular and renal outcomes after coronary angiography. Circulation.

[35] Haase M, Bellomo R, Albert C (2014). The identification of three novel biomarkers of major adverse kidney events. Biomark Med.

[36] Mehta R, Bihorac A, Selby NM (2016). Establishing a continuum of acute kidney injury - tracing AKI using data source linkage and long-term follow-up: workgroup statements from the 15th ADQI Consensus Conference. Can J Kidney Health Dis.

[37] Dewitte A, Joannes-Boyau O, Sidobre C (2015). Kinetic eGFR and novel AKI biomarkers to predict renal recovery. Clin J Am Soc Nephrol.

[38] Chawla LS, Amdur RL, Shaw AD, Faselis C, Palant CE, Kimmel PL (2014). Association between AKI and long-term renal and cardiovascular outcomes in United States veterans. Clin J Am Soc Nephrol.

[39] Bagshaw SM, Laupland KB, Doig CJ (2005). Prognosis for long-term survival and renal recovery in critically ill patients with severe acute renal failure: a population-based study. Crit Care.

[40] Coca SG, Singanamala S, Parikh CR (2012). Chronic kidney disease after acute kidney injury: a systematic review and meta-analysis. Kidney Int.

[41] Kim SM, Cha RH, Lee JP (2010). Incidence and outcomes of contrast-induced nephropathy after computed tomography in patients with CKD: a quality improvement report. Am J Kidney Dis.

[42] Weisbord SD, Mor MK, Resnick AL, Hartwig KC, Palevsky PM, Fine MJ (2008). Incidence and outcomes of contrast-induced AKI following computed tomography. Clin J Am Soc Nephrol.

[43] Davenport MS, Khalatbari S, Cohan RH, Dillman JR, Myles JD, Ellis JH (2013). Contrast material-induced nephrotoxicity and intravenous low-osmolality iodinated contrast material: risk stratification by using estimated glomerular filtration rate. Radiology.

[44] Stacul F, van der Molen AJ, Reimer P (2011). Contrast induced nephropathy: updated ESUR Contrast Media Safety Committee guidelines. Eur Radiol.

[45] Yin WJ, Yi YH, Guan XF et al (2017) Preprocedural prediction model for contrast-induced nephropathy patients. J Am Heart Assoc 6 10.1161/JAHA.116.004498 PMC5523753 28159819

[46] Xu Qian, Wang Na-Na, Duan Shao-Bin, Liu Na, Lei Rong, Cheng Wei, Zhou Shun-Ke (2016). Serum cystatin c is not superior to serum creatinine for early diagnosis of contrast-induced nephropathy in patients who underwent angiography. Journal of Clinical Laboratory Analysis.

[47] van der Molen AJ, Reimer P, Dekkers IA (2018). Post-contrast acute kidney injury. Part 2: risk stratification, role of hydration and other prophylactic measures, patients taking metformin and chronic dialysis patients. Eur Radiol.

[48] Morcos Sameh K., Thomsen Henrik S., Webb Judith A., Contrast Media Safety Committee of members of the (2002). Dialysis and contrast media. European Radiology.

[49] Karlsberg RP, Dohad SY, Sheng R, Iodixanol Peripheral Computed Tomographic Angiography Study Investigator Panel (2011) Contrast medium-induced acute kidney injury: comparison of intravenous and intraarterial administration of iodinated contrast medium. J Vasc Interv Radiol 22:1159–1165 10.1016/j.jvir.2011.03.020 21570871

[50] Kooiman J, Le Haen PA, Gezgin G (2013). Contrast-induced acute kidney injury and clinical outcomes after intra-arterial and intravenous contrast administration: risk comparison adjusted for patient characteristics by design. Am Heart J.

[51] McDonald JS, Leake CB, McDonald RJ (2016). Acute kidney injury after intravenous versus intra-arterial contrast material administration in a paired cohort. Invest Radiol.

[52] Nyman Ulf, Almén Torsten, Jacobsson Bo, Aspelin Peter (2012). Are intravenous injections of contrast media really less nephrotoxic than intra-arterial injections?. European Radiology.

[53] Heinrich MC, Haberle L, Muller V, Bautz W, Uder M (2009). Nephrotoxicity of iso-osmolar iodixanol compared with nonionic low-osmolar contrast media: meta-analysis of randomized controlled trials. Radiology.

[54] Thomsen HS, Morcos SK (2009). Risk of contrast-medium-induced nephropathy in high-risk patients undergoing MDCT--a pooled analysis of two randomized trials. Eur Radiol.

[55] From AM, Al Badarin FJ, McDonald FS, Bartholmai BJ, Cha SS, Rihal CS (2010). Iodixanol versus low-osmolar contrast media for prevention of contrast induced nephropathy: meta-analysis of randomized, controlled trials. Circ Cardiovasc Interv.

[56] Eng J, Wilson RF, Subramaniam RM (2016). Comparative effect of contrast media type on the incidence of contrast-induced nephropathy: a systematic review and meta-analysis. Ann Intern Med.

[57] Zhao F, Lei R, Yang SK (2019). Comparative effect of iso-osmolar versus low-osmolar contrast media on the incidence of contrast-induced acute kidney injury in diabetic patients: a systematic review and meta-analysis. Cancer Imaging.

[58] Castaldo P, Frasca GM, Brigante F (2019). Low incidence of nephrotoxicity following intravenous administration of iodinated contrast media: a prospective study. Eur Radiol.

[59] Sohn KH, Kim GW, Lee SY (2019). Immediate and delayed hypersensitivity after intra-arterial injection of iodinated contrast media: a prospective study in patients with coronary angiography. Eur Radiol.

