# Supplementary material for: Post-contrast acute kidney injury in a hospitalized population: short-, mid-, and long-term outcome and risk factors for adverse events
Source: Eur Radiol. 2020 Feb 21;30(6):3516–27. doi: 10.1007/s00330-020-06690-3 (PMC7248019; doi:10.1007/s00330-020-06690-3)

**Table S1 The clinical, laboratory basic data of PC-AKI with persistent renal dysfunction and transient renal dysfunction**

| **Variables** | **PC-AKI**  **with Transient RD**  **(n=153)** | **PC-AKI**  **with Persistent RD**  **(n=135)** | **P value** |
| --- | --- | --- | --- |
| Age，year | 56.09±12.77 | 55.07±13.81 | 0.516 |
| Age ≥ 65 years, No. (%) | 41 (26.8) | 35 (25.9) | 0.867 |
| Gender (Women), No. (%) | 57 (37.3) | 51 (37.8) | 0.927 |
| Oliguria or anuria, No. (%) | 3 (2.0) | 31 (23.0) | 0.000 |
| X-ray procedures, No. (%) |  |  | 0.006 |
| Enhanced CT | 92 (60.1) | 59 (43.7) |  |
| CT angiography | 38 (24.8) | 57 (42.2) |  |
| PCI | 23 (15.0) | 19 (14.1) |  |
| Type of contrast media (IOCM%), No. (%) | 7 (4.6) | 5 (3.7) | 0.695 |
| Volumes of injected contrast media，ml | 82.78±35.07 | 89.67±52.06 | 0.185 |
| Volumes of injected contrast media, No. (%) |  |  | 0.003 |
| < 50 ml | 12 (7.8) | 9 (6.7) |  |
| ≥ 50 ml, < 100 ml | 96 (62.7) | 60 (44.4) |  |
| ≥ 100 ml | 45 (29.4) | 66 (48.9) |  |
|  |  |  |  |
| Complications of AKI, No. (%) |  |  |  |
| Hyperkalemia | 3 (2.0) | 16 (11.9) | 0.001 |
| Metabolic acidosis | 23 (15.0) | 37 (27.4) | 0.010 |
| Heart failure | 30 (19.6) | 45 (33.3) | 0.008 |
| Respiratory failure | 9 (5.9) | 25 (18.5) | 0.001 |
| Shock | 9 (5.9) | 26 (19.3) | 0.001 |
| Central nervous system failure | 20 (13.1) | 26 (19.3) | 0.153 |
| Gastrointestinal bleeding | 5 (3.3) | 11 (8.1) | 0.071 |
| hypertension | 51 (33.3) | 64 (47.4) | 0.015 |
| diabetes | 27 (17.6) | 24 (17.8) | 0.977 |
| Chronic kidney disease | 25 (16.3) | 17 (12.6) | 0.369 |
| cardiopulmonary bypass surgery | 21 (13.7) | 38 (28.1) | 0.002 |
| Hb, g/l | 111.93±24.66 | 107.96±27.73 | 0.204 |
| Anemia(Hb < 100 g/l) | 31 (20.3) | 43 (31.9) | 0.025 |
| Alb, g/l | 35.88±6.57 | 33.83±6.55 | 0.009 |
| hypoalbuminemia (Alb < 30 g/l), No. (%) | 19 (12.6) | 34 (25.4) | 0.006 |
| TC, mmol/L | 4.13±1.60 | 4.18±1.80 | 0.881 |
| Hyperlipidemia (TC ≥ 6.22 mmol/L), No. (%) | 9 (6.0) | 7 (5.3) | 0.799 |
| Total bilirubin, µmol/L, | 33.86±76.90 | 31.50±55.83 | 0.769 |
| Total bilirubin, No. (%) |  |  | 0.500 |
| 0 < 20 µmol/L | 102 (67.5) | 89 (66.4) |  |
| 20 – 32 µmol/L | 29 (19.2) | 22 (16.4) |  |
| 33 – 101 µmol/L | 10 (6.6) | 12 (9.0) |  |
| 102 – 204 µmol/L | 4 (2.6) | 8 (6.0) |  |
| > 204 µmol/L | 6 (4.0) | 3 (2.2) |  |
| Blood urea nitrogen, mmol/L | 8.47±5.26 | 14.70±9.59 | 0.000 |
| Blood urea nitrogen ≥ 7.14 mmol/L, No. (%) | 73 (47.7) | 109 (80.7) | 0.000 |
| RDW-CV, % | 13.95±2.58 | 14.54±2.67 | 0.063 |
| RDW-CV ≥13.7%, No. (%) | 65 (42.5) | 81 (60.0) | 0.003 |
| PLT，300*10^9^/L | 182.00±94.20 | 156.02±94.46 | 0.022 |
| PLT < 100 or > 300*10^9^ /L, No. (%) | 42 (27.5) | 49 (36.5) | 0.107 |
| The baseline eGFR, mL/min/1.73 m^2^ | 91.10±24.05 | 85.24±22.78 | 0.035 |
| The baseline eGFR, No. (%) |  |  | 0.349 |
| < 60 mL/min/1.73 m^2^ | 16 (10.5) | 19 (14.1%) |  |
| ≥ 60 mL/min/1.73 m^2^ | 137 (89.5) | 116 (85.9%) |  |
| Proteinuria, No. (%) | 30 (19.6) | 36 (26.7%) | 0.155 |
| Acute kidney injury stage, No. (%) |  |  | 0.000 |
| Stage 1 | 153 (100%) | 5 (3.7%) |  |
| Stage 2 | / | 69 (51.1%) |  |
| Stage 3 | / | 61 (45.2%) |  |
| Use of diuretics, No. (%) | 60 (39.2%) | 85 (63.0%) | 0.000 |
| Use of nephroprotective drugs, No. (%) | 13 (8.5%) | 17 (12.6%) | 0.256 |

**Table S2 Risk factors for MAKE30 of PC-AKI patients**

**(Kaplan-Meier analysis-Log Rank)**

| **Variables** | **P value** |
| --- | --- |
| Age ≥ 65 years | 0.759 |
| Gender (Women) | 0.980 |
| X-ray procedures | 0.555 |
| Intravenous CT procedures |  |
|  |  |
| Intraarterial procedure (PCI) |  |
| Type of contrast media | 0.504 |
| Iodixanol |  |
| Iohexol |  |
| Iopromide |  |
| Iopamidol |  |
| Ioversol |  |
| Others |  |
| Volumes of injected contrast media, ml | 0.002 |
| <50 |  |
| ≥ 50, < 100 |  |
| ≥ 100 |  |
| Oliguria or anuria | 0.000 |
| Hyperkalemia | 0.001 |
| Metabolic acidosis | 0.021 |
| Heart failure | 0.005 |
| Respiratory failure | 0.008 |
| Shock | 0.001 |
| Central nervous system failure | 0.977 |
| Gastrointestinal bleeding | 0.428 |
| hypertension | 0.007 |
| diabetes | 0.521 |
| Chronic kidney disease | 0.377 |
| cardiopulmonary bypass surgery | 0.024 |
| Anemia (Hb < 100 g/l) | 0.119 |
| Hypoalbuminemia (Alb < 30 g/l) | 0.007 |
| Hyperlipidemia (TC ≥ 6.22 mmol/L) | 0.602 |
| Total bilirubin, µmol/L, | 0.740 |
| 0 < 20 |  |
| 20 - 32 |  |
| 33 - 101 |  |
| 102 - 204 |  |
| > 204 |  |
| Blood urea nitrogen ≥ 7.14mmol/L | 0.000 |
| RDW-CV ≥ 13.7% | 0.092 |
| PLT < 100 or > 300*10^9^/L | 0.415 |
| The baseline eGFR, mL/min/1.73 m^2^ | 0.097 |
| < 60 |  |
| ≥ 60 |  |
| Proteinuria | 0.004 |
| Acute kidney injury stage | 0.000 |
| Stage 1 |  |
| Stage 2 |  |
| Stage 3 |  |
| Use of diuretics | 0.000 |
| Use of nephroprotective drugs | 0.589 |

**Table S3 Risk factors for 90-day all-cause mortality of PC-AKI patients**

**(Kaplan-Meier analysis-Log Rank)**

| **Variables** | **P value** |
| --- | --- |
| Age ≥ 65 years, No. (%) | 0.206 |
| Gender (Women), No. (%) | 0.076 |
| X-ray procedures | 0.017 |
| Intravenous CT procedures |  |
|  |  |
| Intraarterial procedure (PCI) |  |
| Type of contrast media | 0.065 |
| Iodixanol |  |
| Iohexol |  |
| Iopromide |  |
| Iopamidol |  |
| Ioversol |  |
| Others |  |
| Volumes of injected contrast media, ml | 0.967 |
| <50 |  |
| ≥ 50, < 100 |  |
| ≥ 100 |  |
| Oliguria or anuria | 0.000 |
| Hyperkalemia | 0.001 |
| Metabolic acidosis | 0.000 |
| Heart failure | 0.000 |
| Respiratory failure | 0.000 |
| Shock | 0.000 |
| Central nervous system failure | 0.000 |
| Gastrointestinal bleeding | 0.441 |
| hypertension | 0.057 |
| diabetes | 0.798 |
| Chronic kidney disease | 0.512 |
| cardiopulmonary bypass surgery | 0.515 |
| Anemia (Hb < 100 g/l) | 0.136 |
| Hypoalbuminemia (Alb < 30 g/l) | 0.156 |
| Hyperlipidemia (TC ≥ 6.22 mmol/L) | 0.493 |
| Total bilirubin, µmol/L, | 0.722 |
| 0 < 20 |  |
| 20 - 32 |  |
| 33 - 101 |  |
| 102 - 204 |  |
| > 204 |  |
| Blood urea nitrogen ≥ 7.14mmol/L | 0.000 |
| RDW-CV ≥ 13.7% | 0.069 |
| PLT < 100 or > 300*10^9^/L | 0.629 |
| The baseline eGFR, mL/min/1.73 m^2^ | 0.001 |
| < 60 |  |
| ≥ 60 |  |
| Proteinuria | 0.136 |
| Acute kidney injury stage | 0.002 |
| Stage 1 |  |
| Stage 2 |  |
| Stage 3 |  |
| Use of diuretics | 0.004 |
| Use of nephroprotective drugs or hydration | 0.515 |
| Persistent renal dysfunction (serum creatinine value ≥ 200% the baseline serum creatinine value) | 0.000 |
| Receipt of new RRT within 30 days | 0.000 |

**Table S4 Risk factors for one-year all-cause mortality of PC-AKI patients.**

**(Kaplan-Meier analysis-Log Rank)**

| **Variables** | **P value** |
| --- | --- |
| Age ≥ 65 years, No. (%) | 0.076 |
| Gender (Women), No. (%) | 0.209 |
| X-ray procedures | 0.007 |
| Intravenous CT procedures |  |
|  |  |
| Intraarterial procedure (PCI) |  |
| Type of contrast media | 0.088 |
| Iodixanol |  |
| Iohexol |  |
| Iopromide |  |
| Iopamidol |  |
| Ioversol |  |
| Others |  |
| Volumes of injected contrast media, ml | 0.909 |
| <50 |  |
| ≥ 50, < 100 |  |
| ≥ 100 |  |
| Oliguria or anuria | 0.000 |
| Hyperkalemia | 0.001 |
| Metabolic acidosis | 0.000 |
| Heart failure | 0.000 |
| Respiratory failure | 0.000 |
| Shock | 0.000 |
| Central nervous system failure | 0.000 |
| Gastrointestinal bleeding | 0.404 |
| hypertension | 0.053 |
| diabetes | 0.687 |
| Chronic kidney disease | 0.437 |
| cardiopulmonary bypass surgery | 0.639 |
| Anemia (Hb < 100 g/l)，No. (%) | 0.205 |
| Hypoalbuminemia (Alb < 30 g/l), No. (%) | 0.211 |
| Hyperlipidemia (TC ≥ 6.22 mmol/L), No. (%) | 0.533 |
| Total bilirubin, µmol/L | 0.707 |
| 0 < 20 |  |
| 20 - 32 |  |
| 33 - 101 |  |
| 102 - 204 |  |
| > 204 |  |
| Blood urea nitrogen ≥ 7.14mmol/L | 0.000 |
| RDW-CV ≥ 13.7% | 0.074 |
| PLT < 100 or > 300*10^9^/L |  |
| The baseline eGFR, mL/min/1.73 m^2^ | 0.002 |
| < 60 |  |
| ≥ 60 |  |
| Proteinuria | 0.095 |
| Acute kidney injury stage, No. (%) | 0.000 |
| Stage 1 |  |
| Stage 2 |  |
| Stage 3 |  |
| Use of diuretics | 0.011 |
| Use of nephroprotective drugs | 0.585 |
| Persistent renal dysfunction (serum creatinine value ≥ 200% the baseline serum creatinine value) | 0.000 |
| Receipt of new RRT within 30 days | 0.000 |

**Table S5 Effect of diuretics or nephroprotective drugs on prognosis of PC-AKI patients**

| Drugs | No. (%) of  PC-AKI Patients | P value (Log Rank method) | | |
| --- | --- | --- | --- | --- |
|  |  | MAKE30 | 90-day mortality | 1-year mortality |
| Nephroprotective drugs | | | | |
| Prior | 26 (9.0) | 0.894 | 0.310 | 0.360 |
| After | 18 (6.3) | 0.056 | 0.574 | 0.624 |
| Prior and after | 14 (4.9) | 0.187 | 0.279 | 0.309 |
| Prior or after | 30 (10.4) | 0.589 | 0.515 | 0.585 |
| Diuretics | 145 (50.3%) | 0.000 | 0.004 | 0.011 |

Figure S1


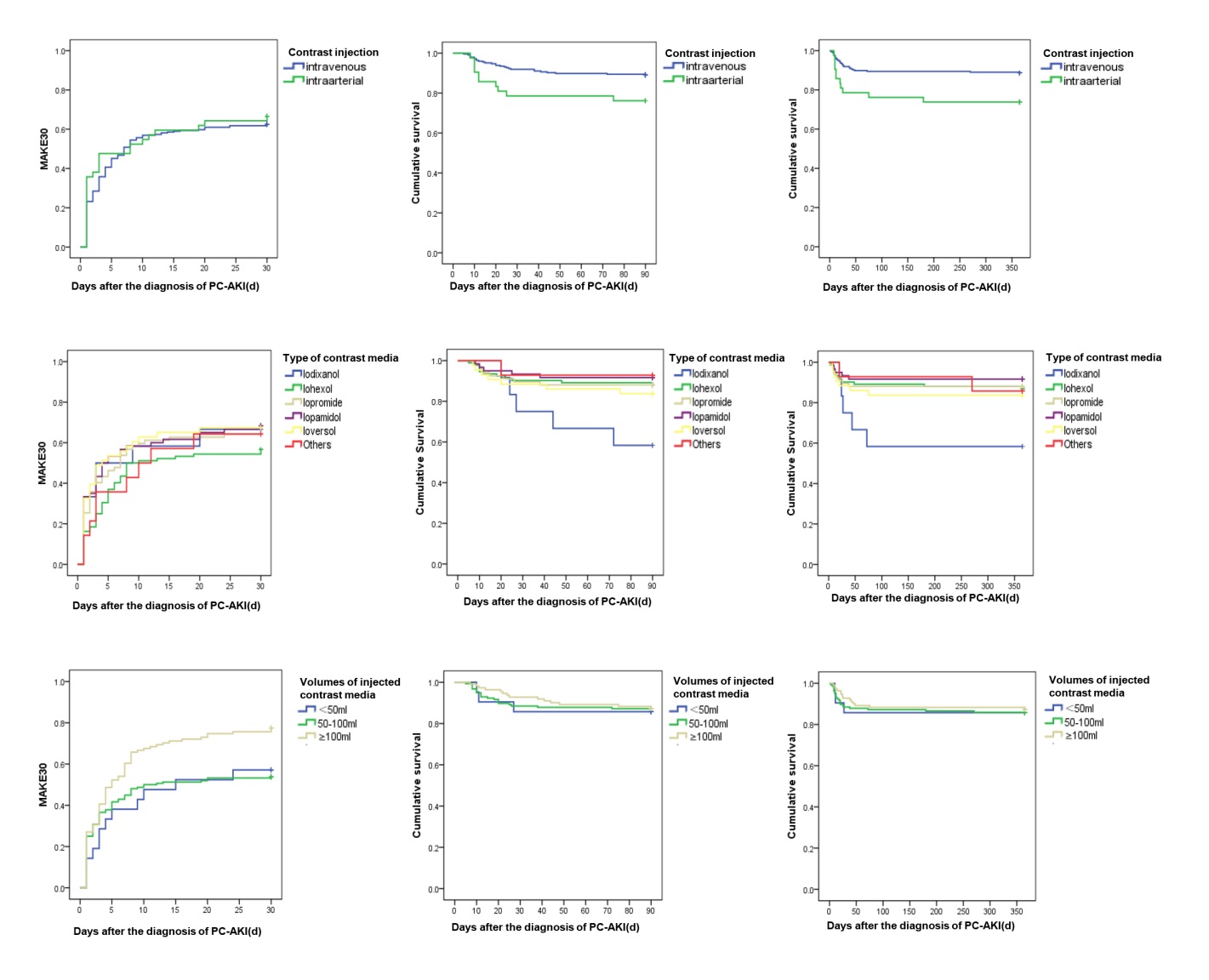


Figure S2


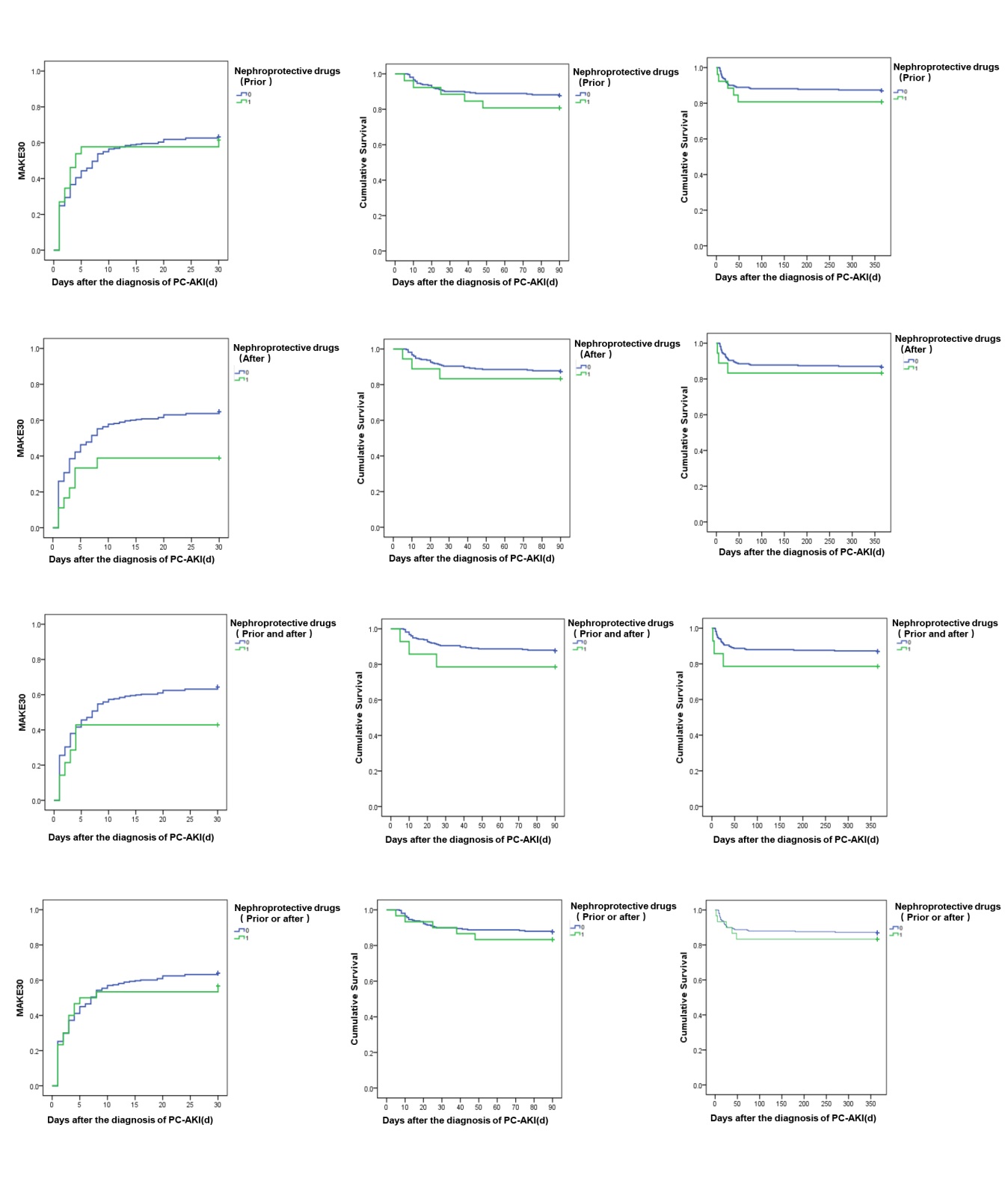

Supplement: Supplementary file 1 — (DOCX 455 kb) [file 330_2020_6690_MOESM1_ESM.docx]
