# Supplementary figures and images for: Post-contrast acute kidney injury in a hospitalized population: short-, mid-, and long-term outcome and risk factors for adverse events
Source: Eur Radiol. 2020 Feb 21;30(6):3516–27. doi: 10.1007/s00330-020-06690-3 (PMC7248019; doi:10.1007/s00330-020-06690-3)

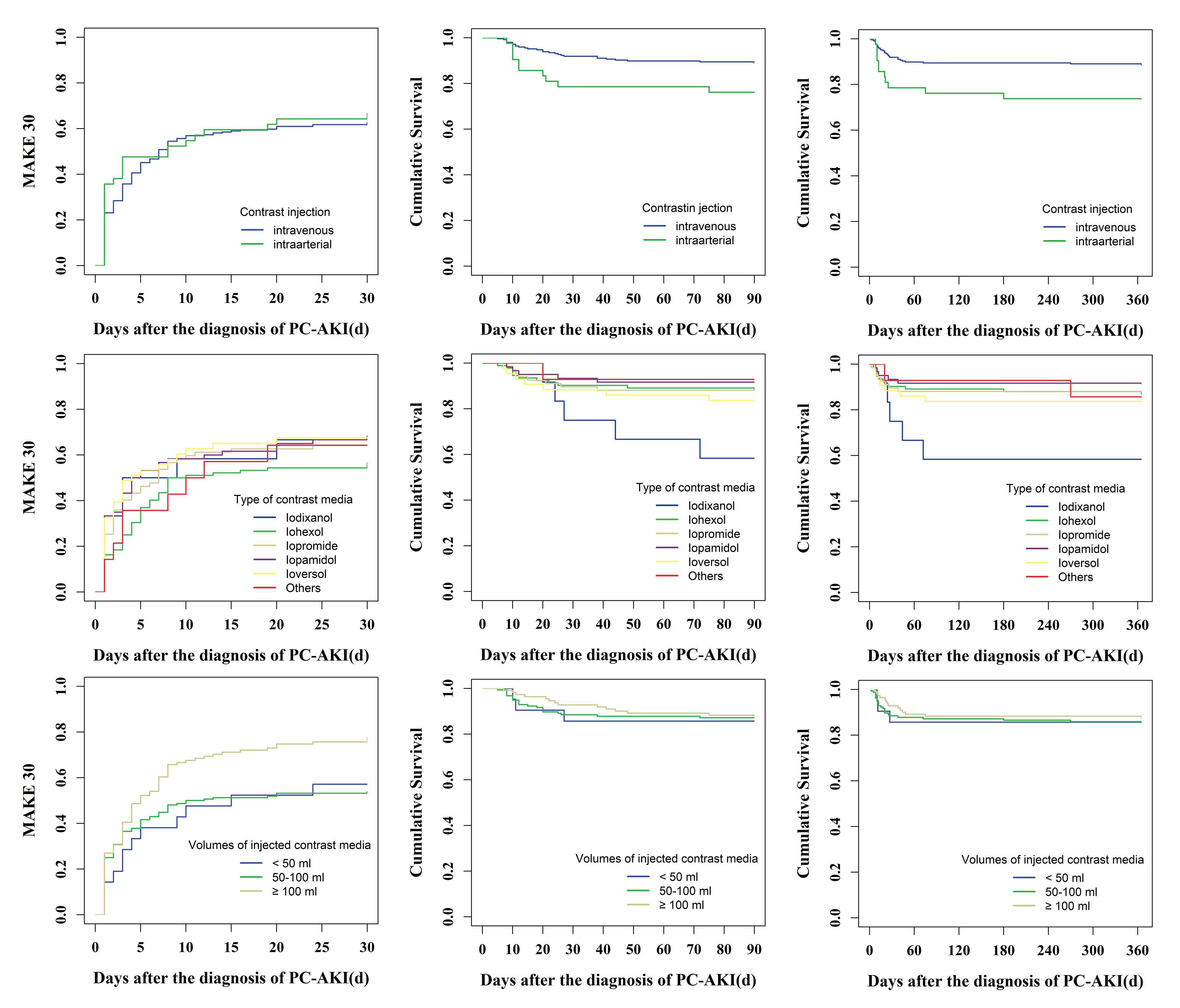

Supplement: Supplementary file 2 — (TIF 1.44 MB) [file 330_2020_6690_MOESM2_ESM.tif]

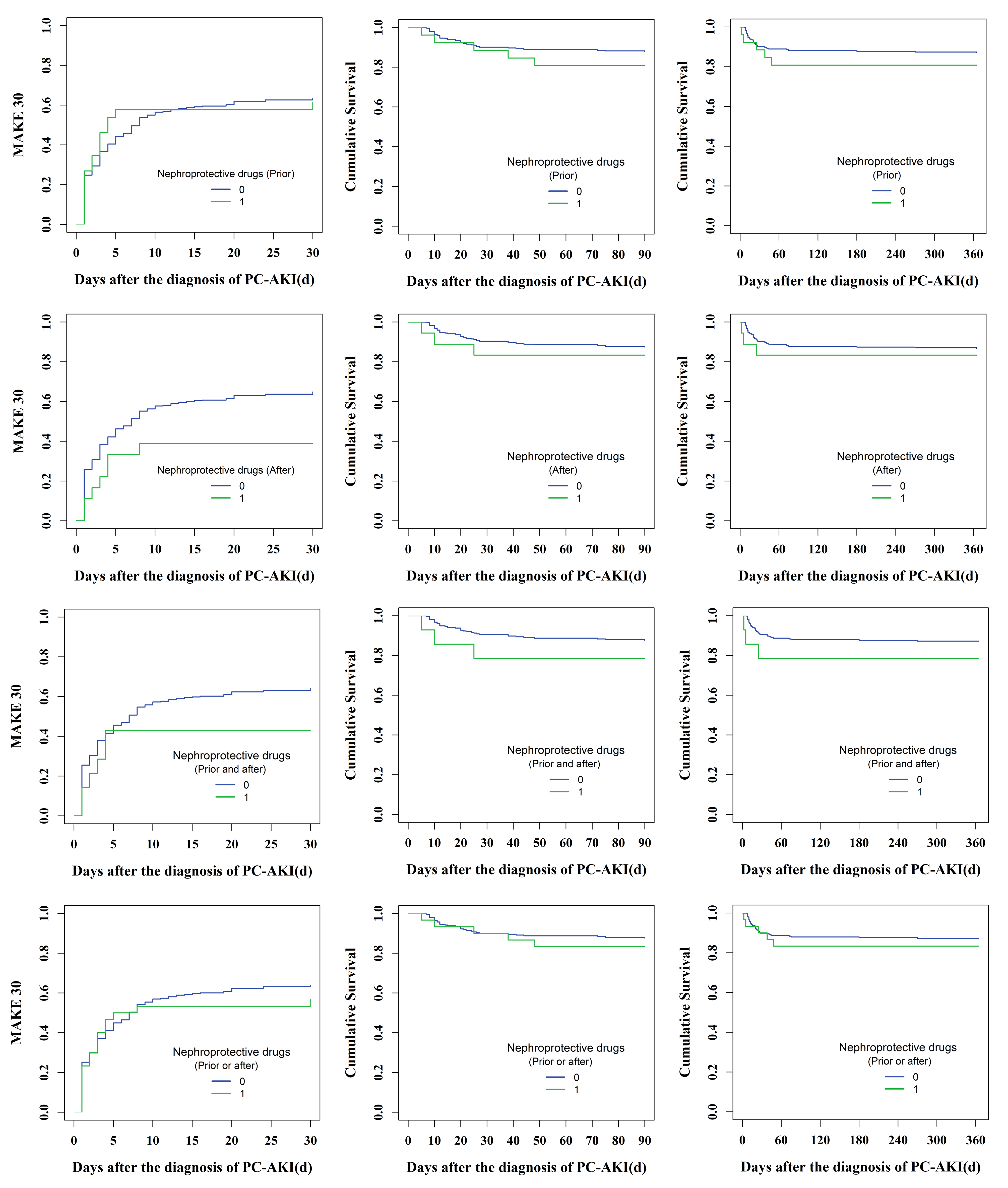

Supplement: Supplementary file 3 — (TIF 1.72 MB) [file 330_2020_6690_MOESM3_ESM.tif]
